# Optimal equity capital requirements for large Swiss banks

**DOI:** 10.1186/s41937-018-0025-z

**Published:** 2018-08-22

**Authors:** Georg Junge, Peter Kugler

**Affiliations:** 1 Risk Consulting and Partner, Arabienstrasse 28, CH-4059 Basel, Switzerland; 2 Faculty of Business and Economics, University of Basel, Peter Merian-Weg 6, CH-4002 Basel, Switzerland

**Keywords:** Financial regulation, Bank equity capital requirements, Capital structure, Elasticity of substitution, Translog production function, G21, G28, E20, E22

## Abstract

Ten years after the worst financial crisis of the post-war period, Switzerland has established a Too-Big-To-Fail (TBTF) framework. Under this framework, the two large Swiss banks are subject to substantial capital requirements. It is not obvious whether the TBTF capital requirements are sufficient to prevent banks from plunging the country into a financial crisis once again. We estimate the social costs and benefits of higher capital requirements for the two large Swiss banks and derive socially optimal capital ratios from the cost-benefit trade-off. Our results show that Swiss TBTF capital requirements still fall short of socially optimal capital ratios.

## Introduction

This paper seeks to contribute to the discussion of the optimal equity capital requirements for banks from a society’s perspective. In Junge and Kugler (2013), we limited ourselves to a comparison of the social costs and benefits and concluded that long-run benefits exceed long-run costs by a substantial multiple.^1^ In this paper, we present an attempt to determine the optimal leverage and capital ratios for Switzerland’s global systemically important banks (G-SIBs).

The economic debate about the appropriate minimum level of regulatory capital requirements for banks from society’s perspective is highly controversial. At one end of the spectrum, Admati and Hellwig (2013, p. 179) argue that there are no social costs associated with higher equity capital requirements and propose a leverage ratio requiring equity capital on the order of 20 to 30% of total assets. At the other end, banking industry representatives continue to emphasize that higher equity capital requirements in particular reduce the availability of credit and retard economic growth.^2^ The conflict over the appropriate minimum level of banking capital also blocked the finalization of Basel III at the beginning of 2017. Some members of the Basel Committee on Banking Supervision (BCBS) emphasized that only strongly capitalized and highly liquid banks can support economic growth, while others argued that the pendulum of the Basel III revisions had already swung too far and undermined the economic recovery.^3^

In October 2015, Switzerland amended its Too-Big-To-Fail (TBTF) legislation and decided to raise the required going concern leverage ratio for Switzerland’s G-SIBs—Credit Suisse and UBS—to 5%.^4^ This decision was based on the recommendation of the “Group of Experts on the Further Development of the Financial Market Strategy in Switzerland” that Switzerland should be among the countries with the most stringent capital requirements.^5^ Designing Swiss capital requirements along the same lines as foreign standards is one choice, as well as the orientation on international competitiveness.^6^ However, as relevant as they are, these considerations should be complementary in nature as they do not address the key question of whether the new TBTF capital requirements are appropriate from society’s point of view. An optimal level of bank equity capital should be determined by some aggregate welfare objective taking into account that higher equity capital requirements benefit the economy by reducing the likelihood of banking crises while simultaneously imposing economic cost in terms of a lower potential economic output. Along these lines, we extend Junge and Kugler (2013) and seek to determine the long-run steady-state optimal leverage and capital ratios for the Swiss G-SIBs.^7^ This approach is in accordance with a major strand of economic research on bank capital and regulatory requirements. After the financial crisis of 2007/2008, the approach was applied by, among others, the BCBS (2010a), Kashyap et al. (2010), and Miles et al. (2011 and 2013).^8^

Our article is arranged as follows. In Section 2 we present an updated estimate of the size of the Modigliani-Miller (M-M) effect for Switzerland’s G-SIBs, extending the sample period of Junge and Kugler (2013) by 5 years up to 2015. Based on the M-M effect, we calculate in Section 3 the banks’ overall cost of funds and the social cost of higher equity capital requirements using a translog production function. In Section 4 we re-estimate the effect of banking crises using a novel and extensive data set from 1892 to 2016 and combine this with the analysis of Junge and Kugler (2013) to obtain a social benefit curve for additional equity capital requirements. In Section 5 we compare the social cost and benefit associated with higher equity capital requirements and determine optimal leverage and capital ratios under different capital definitions. Finally, Section 6 concludes.

## Empirical evidence of the Modigliani-Miller theorem of capital structure irrelevance for Swiss G-SIBs

As shown by Modigliani-Miller, a company’s overall cost of funds is unaffected by the mix of equity and debt under perfect capital markets and in the absence of taxes and subsidies. An increase in equity, which is more expensive than debt, will simply be offset by a new mix of equity and debt with lower required rates of return on equity and debt.^9^ In this case, the banks’ overall funding costs will not change and therefore the lending of banks will remain unaffected. However, if the idealized conditions of the M-M theorem are not perfectly met, the M-M offset is incomplete and an increase in equity will raise the funding costs and consequently bank lending rates. The key empirical question is to what extent the mechanism holds for the Swiss G-SIBs.

The estimation of the size of the M-M offset was first explored by Kashyap et al. (2010) as well as Miles et al. (2011 and 2013) and was applied to Swiss data by Junge and Kugler (2013). The framework is derived from the Capital Asset Pricing Model (CAPM) and the M-M theorem. Assuming that bank debt is risk free, the following linear relationship between systematic equity risk and leverage is obtained^10^:

1 $$ {\beta}_{\mathrm{equity}}={\beta}_{\mathrm{asset}}\ \frac{E+D}{E} $$

where *β*_equity_ is the systematic equity risk of the bank, *β*_asset_ is the systematic risk on the bank’s assets, and $Lev=\frac{E+D}{E}$ is the bank’s leverage with its equity (*E*) and debt (*D*) components.

According to Eq. (1), a reduction in leverage (i.e., a relative increase in equity) leads to a proportional decline of systematic equity risk. For example, assume a bank initially has a leverage of 40 and an equity market beta of 2. If equity is doubled, and hence leverage is halved to 20, equity beta declines from 2 to 1.

As pointed out in a recent study by Clark et al. (2015),^11^ Eq. 1 is an appropriate specification for TBTF banks that benefit from implicit government guarantees and from deposit insurance in general. In this situation, the market perceives the debt of TBTF banks as risk free and the adjustment to changes in leverage will be channeled through equity as stated in Eq. (1).^12^ In contrast, for smaller, non-TBTF banks, the debt mechanism for adjustment cannot be ignored and the present framework is less appropriate.

Equation (1) can be tested directly by running a regression of *β*_equity_ on leverage and testing the hypothesis that the intercept is equal to zero (a 100% M-M offset). Alternatively, we can generalize Eq. (1) by considering the log-linear model *β*_equity_ = *β*_asset_*L*^*b*^ and test the 100% M-M hypothesis that *c* is equal to 1. The intercept term of this regression is now log(*β*_asset_) and should have a negative sign. Both tests are performed below.

In our 2013 study, we employed quarterly data from 1999 and 2010 and estimated an M-M offset of 55% (log-linear) for the two Swiss G-SIBs. But much has happened since 2010. In response to the Swiss TBTF legislation, both banks have more than doubled their common equity (CET1) levels. In mid-2015, Credit Suisse reported a CET1 ratio of 10.3% of risk-weighted assets and UBS a ratio of 14.4% which can be compared to a benchmark of 4.5% of RWA at the end of 2010.^13^ In addition, both banks enhanced their liquidity ratios and are in the process of implementing the TBTF resolution requirements.

Table 1 reports the results of linear and log-linear regressions of equity beta (estimated in the framework of the CAPM) on lagged bank leverage. Lagged bank leverage is used as a regressor in order to avoid potential endogeneity problems. The panel characteristic of the data is taken into account by fixed bank effects as well as a fixed or random time effect. The random time effect model is adopted in order to get an efficiency gain in estimation when the Hausman test shows no significant correlation of the regressor and the time effects.

**Table 1:** Bank equity beta and bank leverage

	Linear	Log-linear	Linear	Log-linear	Linear	Log-linear
Frequency	Quarterly	Quarterly	Quarterly	Quarterly	Quarterly	Quarterly
Time period	2001Q2–2015Q2	2001Q2–2015Q2	2001Q2–2009Q4	2001Q2–2009Q4	2010Q2–2015Q2	2010Q2–2015Q2
No. of observations	57	57	35	35	21	21
Bank effect	Fixed	Fixed	Fixed	Fixed	Fixed	Fixed
*F*-statistics	12.2225^***^	17.7367^***^	9.5651^***^	15.1629^***^	4.5103^**^	4.9486^**^
Time effect	Fixed	Fixed	Fixed	Fixed	Random	Random
*F*-statistic	5.7657^***^	5.7033^***^	5.5793	15.1629^***^	–	–
*a* (constant)	0.8269^***^ (0.1605)	− 1.5512^***^ (0.4225)	0.6778^***^ (0.2148)	− 1.7322^***^ (0.5025)	0.6753^**^ (0.3093)	− 1.7731^**^ (0.7218)
*b*	0.01754^**^ (0.003904)	0.5340^***^ (0.1157)	0.01832^***^ (0.00471)	0.5551^***^ (0.1157)	0.02920^***^ (0.01007)	0.6487^***^ (0.2188)
*t* statistic H0: *b* = 1	–	4.0277^***^	–	3.8543^***^	–	1.6056
Adj. *R*-squared	0.7132	0.7132	0.7001	0.6903	0.1031	0.1437
Hausman test	5.0166^**^	7.3111^***^	4.8945^**^	8.6245^***^	2.4799	2.0465
Mean beta	1.54		1.57		1.49	
Mean leverage	40.38		48.47		27.18	
Gauging the M-M effect of the linear regression	0.460		0.566		0.533	

Note: ^*^,^**^,^***^Significance at the 5%, 1%, and 0.1% level, respectively (null hypothesis for *t* statistics: *b* = 0, *a* = 0). Standard errors are given in parentheses. Definition and sources: The beta estimates for Credit Suisse and UBS are obtained by regressing each quarter of the bank’s daily stock returns on the daily return of the Swiss performance Index (SPI). Daily closing prices for the banks and the SPI are obtained from FactSet. Leverage is defined as the ratio of balance sheet to BIS Tier 1 capital. Balance sheet assets and BIS Tier 1 capital are collected from Bloomberg and the bank’s quarterly reports at group level

Table 1 shows the estimates for the full sample and a sample split in 2010. For the full sample, we have to adopt the two-way fixed effects model because bank as well as time effects are highly statistically significant and appear to be correlated with the residuals (according to the Hausman test).

The estimates of the log-linear model are highly statistically significant with a slope coefficient of 0.534 which is very close to the value reported by Junge and Kugler (2013). This estimate is significantly below 1 and therefore points to a partial M-M offset. As to the linear regression, we notice a positive and significant intercept and a significant slope coefficient of 0.0175 which implies an elasticity (M-M offset) of 0.46 evaluated at the means of beta and leverage.^14^ A significant intercept again rejects the hypothesis of a full M-M offset and confirms the existence of a partial M-M offset. The estimates for the first sub-period until 2010 are very close to those of the full sample. For the second sub-sample, the slope coefficient is larger than the first sub-sample, namely 0.0292 implying an elasticity (M-M offset) of 0.533 at the means, whereas the directly estimated log-linear elasticity is 0.649. The first sub-sample estimate is within one standard error of the second sub-sample estimate, and we find, therefore, no sign of a structural break in the regressions. Note that we could use the random time effect specification in the second sub-period according to the Hausman test. Moreover, the sizably lower adjusted *R*-squared in the random effect model is to be expected, as the time dummy variables in the fixed effect model contribute to the *R*-squared whereas in the random effects model these effects are in the error term.

In summary, the results of Table 1 not only confirm our earlier findings, they also show that the M-M offset for the Swiss TBTF banks is robust across sub-periods and sizeable, amounting to about 50% of what is predicted under full M-M validity. This applies equally to the linear and the log-linear specification of the regression. Particularly important is the stability of the size of the M-M offset given the changes in regulatory and economic conditions for the Swiss G-SIBs after 2010. This evidence is in line with other studies that find M-M offsets in the range of 40 to 70%.^15^

## Social cost of additional capital requirements

### Bank funding costs

As already mentioned, if the M-M offset is incomplete as in the case of the Swiss G-SIBs, higher equity requirements will increase the funding cost of banks. The banks will pass on the additional cost to borrowers, and bank lending rates will rise. This in turn raises the economic costs of capital formation and leads ultimately to a permanent drop in GDP. In our model, the banks’ funding costs are the weighted average cost of capital, *WACC*. As we assume that debt has a zero beta, the cost of debt is equal to the risk-free rate *R*_*f*_. Given these assumptions, the banks’ *WACC* is:

2 $$ WACC(LR)={R}_{\mathrm{Equity}}\frac{E}{D+E}+{R}_f\left(1-\frac{E\ }{D+E}\right), $$

where *R*_Equity_ is the expected return on equity and *R*_*f*_ the risk-free rate. $\frac{E}{D+E}$ is the leverage ratio (LR). Since we estimated the size of the M-M effect as a function of leverage (rather than the leverage ratio), we rearrange Eq. (2) in terms of leverage. For this, we replace $\frac{E}{E+D}$ by $\frac{1\ }{L}$, where *Lev* stands for leverage.

3 $$ WACC(Lev)={R}_{\mathrm{Equity}}\frac{1}{Lev}+{R}_f\left(1-\frac{1\ }{Lev}\ \right)=\kern0.5em {R}_{\mathrm{Equity}}\frac{1}{Lev}-{R}_f\frac{1\ }{Lev}+{R}_f\kern0.5em $$

In line with the leading empirical studies of the M-M offset,^16^ we apply the CAPM in order to include the results of our regressions between leverage and *β*_equity_ in Eq. (3). The CAPM states that the required return on equity, *R*_Equity_, is proportional to the (bank specific) beta, *β*_equity_, times the equity market risk premium, *R*_*p*_ .

4 $$ {R}_{\mathrm{Equity}}={R}_f+{\beta}_{\mathrm{Equity}}\bullet {R}_p={R}_f+\left(\widehat{a}+\widehat{b}\  Lev\right){R}_p $$

where $\widehat{a}$ is the constant and $\widehat{b}$ is the coefficient on leverage from our beta regressions (see Table 1).

Substituting Eq. (4) into Eq. (3) yields:

5 $$ WACC(Lev)={R}_f+\left(\frac{\widehat{a}}{Lev}+\widehat{b}\right){R}_p $$

Equation (5) shows that *WACC* is an inverse function of leverage and depends on the regression estimates $\widehat{a}$ and $\widehat{b}$. These coefficients are based on Pre-Basel III definitions of leverage, i.e., of the ratio of Balance Sheet Assets to BIS Basel II Tier1 capital. In order to express *WACC* in terms of the definitions of the Basel III Accord, we need to convert the Pre-Basel III definition of leverage accordingly. Assuming that *C*_con_ is the conversion factor between the Pre-Basel III and the Basel III definition of leverage, we write Eq. (5) as follows:

6 $$ WACC\left({Lev}_{\mathrm{Basel}\_\mathrm{III}}\right)={R}_f+\left(\frac{\widehat{a}}{C_{\mathrm{con}}\bullet {Lev}_{\mathrm{Basel}\_\mathrm{III}}}+\widehat{b}\right){R}_p $$

Equation (6) includes all the elements needed to calculate the overall funding cost of the Swiss G-SIBs, which can be rewritten in terms of the leverage ratio as:

7 $$ WACC\left(L{R}_{\mathrm{Basel}\_\mathrm{III}}\right)={R}_f+\left(\frac{\widehat{a}\bullet L{R}_{\mathrm{Basel}\_\mathrm{III}}}{C_{\mathrm{con}}}+\widehat{b}\right){R}_p $$

Thus, *WACC* is a linear function of the leverage ratio. Since $\widehat{a}$ and $\widehat{b}$ are positive, higher capital requirements imply higher cost of capital. The conversion factor *C*_con_ ensures that the leverage ratio is expressed in terms of Basel III Look-through (fully-applied) leverage ratio. Appendix 1 explains in detail the different definitions of the leverage ratio and the derivation of the conversion factors.

In the base case of the calculations developed below, $\dot{\widehat{a}}$ = 0.8269 and $\widehat{b}$ = 0.01754 are the estimated regression coefficients over the sample from 2001 and 2015. The conversion factor *C*_con_ is 0.713 (= 0.77/1.08, see Appendix 1). For risk-free money market rate *R*_*f*_, we use the repo reference rate of the SNB, which was about 1% during this period. For the equity market risk premium, *R*_*p*_, we assume a lower (5%) and an upper (10%) level to take account of the well-known fact that equity risk premiums vary greatly in size over time. All parameters and their values used in our analysis are summarized in the tables of Appendix 3.

Table 2 shows the increase in *WACC* for the Swiss G-SIBs caused by a 1 percentage point increase in the leverage ratio. Two basic scenarios are compared: (i) the estimated M-M offset on *WACC* resulting from the linear regression 2001Q2 to 2015Q2 and (ii) the *WACC* impact under the assumption that the required return remains invariant to leverage, i.e., there is no M-M offset. Moreover, all calculations show the *WACC* before and after conversion to the final Basel III standards as of 1 January 2018. Thus, results are expressed in terms of the Basel III Tier1 Look-through (fully-applied) and CET1 Look-through (fully-applied) definition of the leverage ratio.

**Table 2:** Swiss G-SIBs: impact on *WACC* resulting from a 1 percentage increase of the leverage ratio measured in bps

Leverage ratio	Linear regression (1)		No M-M effect (2)	
	Impact on WACC (RP = 5%)	Impact on WACC (RP = 10%)	Impact on WACC (RP = 5%)	Impact on WACC (RP = 10%)
Pre-Basel III	4.1	8.3	7.7	15.4
Basel III Tier 1 Look-through	5.8	11.6	10.8	21.5
Basel III CET1 Look-through	7.4	14.9	13.8	27.6

Note: (1) The calculation is based on the estimated M-M offset resulting from the linear regression 2001Q2 to 2015Q2 (see Table 1). (2) Calculated under the assumption that the required return remains invariant to leverage

Table 2 confirms the following observations already made in Junge and Kugler (2013):

1. The M-M effect matters. Comparison of the *WACCs* calculated on the basis of the empirically observed M-M effect (left-hand side of Table 3) with those calculated under the assumption of no M-M validity (right hand section of Table 3) shows that the M-M effect reduces the *WACC* increase by 46%.
2. Not surprisingly, the new more stringent capital requirements under Basel III imply systematically higher *WACCs* compared to Pre-Basel III levels. They are about 40% (for Tier1) and 80% (for CET1) higher than the corresponding pre-Basel III *WACCs*.
3. Increases in the leverage ratio lead to proportional changes in *WACC*. A 1 percentage increase of the leverage ratio raises the Basel III Tier1-based (Look-through) *WACC* by only 5.8 bps (assuming an equity premium of 5%) and by 11.6 bps (assuming an equity premium of 10%). The corresponding *WACCs* for Basel III CET1 are higher amounting to 7.4 and 14.9 bps, respectively.

**Table 3:** Corporate loans G-SIBs versus other banks

Ratio of credit lines to utilization by company size (exposure-weighted averages 2002–2015)				
Company size (no. of employees)	Up to 9	10–49	50–249	Above 249
G-SIBs	1.33	1.43	1.65	1.86
Other banks	1.14	1.20	1.26	1.60

Source: Own calculations based on SNB: corporate loans, broken down by company size: https://data.snb.ch/de/topics/banken#!/cube/bakredbetgrbm. Note: 168 number of observations

### The responsiveness of GDP to the banks’ cost of capital

The starting point is the simple approach adopted by Miles et al. (2011 and 2013), which is based on a production function for GDP with capital (*K*) and labor (*L*) inputs and technological progress represented by a time trend *Y = f*(*K*,*L*,*t*). If factor prices are equal to marginal products, the elasticity of output with respect to the price of capital can be written simply as a function of the substitution elasticity *σ*_*KL*, *t*_ and the elasticity of output with respect to capital *S*_*K*, *t*_ (equal to the income share of capital). The subscript (*t*) reflects the possibility that the elasticity of output, *E*_*Y*, *PK*, *t* _, with respect to the price of capital, *P*_*K*, *t*_, as well as *σ*_*KL*, *t*_ and *S*_*K*, *tt*_ can change over time:

8 $$ \frac{d{Y}_t}{d{P}_{K,t}}\ \frac{P_{K,t}}{Y_t}=-{\sigma}_{KL,t}\frac{S_{K,t}}{1-{S}_{K,t}}=-{E}_{Y, PK,t\kern1.5em } $$

Equation (8) is based on growth theory and therefore provides an estimate of the long-run steady-state impact of an increased price of capital on output. In line with the neoclassical growth theory, a permanent increase in the price of capital alters the equilibrium capital stock and leads to a permanent decline in the level of output as measured by GDP. This is an economy-wide framework, which includes all goods and services produced in the economy and allows us to calculate the economic cost of higher capital cost in terms of lost output.^17^

In Junge and Kugler (2013), we adopt the CES production function with constant *σ* and *S*_*K*_ and estimated an elasticity of substitution between capital and labor for the real (non-financial) sector of approximately 1, as in the special case of the Cobb Douglas production function. This is surprising given the estimates for other advanced countries which are usually clearly lower than one. Moreover, new statistics for Switzerland’s capital stock and the income distribution between capital and labor for the period 1995 to 2014 have recently been published, which provide an opportunity to check the case in a more flexible translog framework. The translog framework is based on a second-order Taylor approximation of an unspecified logarithmic production function. This allows for a time-varying rate of substitution and production elasticity of capital and includes the Cobb Douglas function as a special case.^18^

The estimation of the translog production function is reported in detail in Appendix 2. It results in an elasticity of substitution varying between 0.42 and 0.44 during the period 1995–2014. Together with the time series of the capital cost share (*S*_*K*, *t*_) and the elasticity of substitution (*σ*_*KL*, *t*_), we are able to calculate a time-varying estimate for the elasticity of production with respect to the price of capital as given in Eq. (8), i.e., *E*_*Y*, *PK*, *t* _. This crucial parameter for our analysis varies between 0.27 and 0.34 with a mean and median of approximately 0.31. This implies that the median level of GDP decreases permanently by 0.31% if the cost of capital of non-financial corporations increase by 1%. Interestingly, this parameter reaches its absolute maximum before the financial crisis and decreases in absolute value since 2008, implying a weaker reaction of GDP to capital costs changes in recent years (see Appendix 2, Fig. 6). The result is driven by a decrease in the cost share of capital in production, which is probably the consequence of the increased importance of human capital and skilled labor in production in the last 10 years.

The translog production elasticity of 0.31 lies clearly below the estimate of 0.43 used in Junge and Kugler (2013). However, compared to other advanced countries, the production elasticity of 0.43 appears high. Miles et al. (2011 and 2013) and Clark et al. (2015) apply a production elasticity of 0.25 on the basis of empirical studies related to the UK and USA.^19^ The advantage of our new translog estimate is that it is more plausible than the CES estimate and of the same order of magnitude as the UK and US estimates.

As a next step, we need to determine the capital costs for the Swiss companies in line with the assumed market risk premiums of 5% and 10%. To this end, we estimate the equity beta of Swiss non-financial companies ${\beta}_t^{\mathrm{Corp}}$

9 $$ {r}_t^{\mathrm{Corp}\_\mathrm{SPI}}={\alpha}_t+{\beta}_t^{\mathrm{Corp}}\cdot {r}_t^{\mathrm{SPI}}+{\epsilon}_t $$

where ${r}_t^{\mathrm{Corp}\_\mathrm{SPI}}$ is the log return on the corporate sector of the SPI index (i.e., excluding financial and insurance companies) and ${r}_t^{\mathrm{SPI}}$ is the log return on the SPI.

Not surprisingly, the beta for Swiss non-financial companies, *β*^Corp^ turns out to be slightly above 1, namely 1.1. Next, we apply the CAPM and calculate the capital costs for the Swiss non-financial companies,*P*_*K*_, under the same assumptions that are used to calculate the return on equity for the banks. Given the two risk premiums (5% and 10%), we determine a lower (6.5%) and an upper (12%) estimate of the capital cost for Swiss non-financial companies.

As the Swiss TBTF legislation applies to the Swiss G-SIBs, only these institutions are under pressure to increase lending rates.^20^ Consequently, economy-wide lending rates will increase only by a certain proportion, determined by the role of the G-SIBs in credit supply. Since in our approach the impacts of higher *WACCs* are channeled through the Swiss corporate sector, the relevant market share is the share of G-SIBs in external financing of the Swiss corporate sector. This share is 10.8%.^21^

Finally, we assume that any rise in *WACC* is, at least partially, passed on to their customers whereby we distinguish between two simple scenarios: a 100% and a 50% pass through (*PT*). The size of the *PT* depends on the market power of the two G-SIBs reflected in their competitive position and their ability to bind their customers.

A 100% *PT* combined with the assumption that borrowers will not substitute away from UBS and CS to other Swiss banks suggests a perfectly inelastic demand for credit from the two large Swiss banks. Given the fierce competition among Swiss banks, this seems to be rather unlikely at first sight. However, on closer inspection, one notices that banks have certain means to lock-in their customers. In credit markets, banks retain their customers through contingent lending arrangement in the form of credit lines or revolving loans. Credit lines and revolving loan agreements are of considerable value for corporates as it allows them to choose when and how much to borrow as well as to repay loans in line with their business needs. This provides corporates with a great deal of flexibility. A look in the statistics (Table 3) shows that the two Swiss G-SIBs are especially generous in providing credit lines to small and large businesses. The ratios of credit line to utilization provided by the two G-SIBs are always higher than those of the other banks. This is valid on average and for any company size (Table 3).

Next, a bank loan is often part of a wider business relationship between the main (house) bank and a company. It includes for instance, accepting deposits, payment services, check clearing, investment advice, cross selling, and a range of other services.^22^ Typically, these arrangements are close and long-lasting and not easily questioned. They are quite common in Switzerland, Germany, and Austria. It fits this picture that 75% of the Swiss small and medium enterprises (SMEs) have a financing relationship with only one bank. Another 19% have two as shown in a recent survey.^23^ The same survey finds that only 2 to 3% of Swiss SMEs have changed their house bank over the past 12 months or might consider changing it in the next 12 months. Therefore, it is not surprising that the survey concludes that Swiss SMEs are generally satisfied with their house bank and therefore see no reason to change.

From these considerations, we conclude that, despite fierce competition, the two Swiss G-SIBs have limited scope to raise their bank lending rates without borrowers leaving them in masse. In the light of a capital cost increase of between 12 and 22 bps (100% *PT*), we believe that the assumption of an inelastic demand for credit for the two large banks is acceptable. However, in order to investigate the impact of an incomplete pass through, we will include in our calculations a *PT* of 50% and report its impact on the optimal leverage ratio.

The ingredients of the above discussion can be summarized by the GDP multiplier (GDPM) in Eq. (10).

10 $$ \mathrm{GDPM}=\frac{E_{Y, PK,t}\bullet SEF\bullet PT}{R_f+\left({R}_P\bullet {\beta}_{\mathrm{Corp}}\right)}=\frac{E_{Y, PK,t}\bullet SEF\bullet PT}{P_{K,t}}\kern0.75em $$

Equation (10) states that the responsiveness of output depends on the elasticity of production with respect to the price of capital, *E*_*Y*, *PK*, *t*_ , on the share of external financing of the Swiss corporate sector by the G-SIBs, *SEF*, the percent of the pass through, *PT*, and the price of capital for the Swiss non-financial companies, *P*_*K*, *t*_. As an example, take an increase of *WACC* by 11.6 bps (see Table 2, Basel III Tier1) with 100% *PT*. At a given *SEF* of 10.8%, the cost of capital for the non-financial firms rises by 1.25 bps above its current cost *P*_*K*, *t*_ of 1200 bps. This is an increase of 0.104% (1.25/1200 = 0.104%) and translates into a permanent fall in output of 3.2 bps given the elasticity *E*_*Y*, *PK*, *t*_ of 0.31 (0.31 × 0.104% = 3.2 bps).

Given Eqs. (7) and (10), the GDP cost of higher leverage ratios, *LR*_Basel _ III_ is:

11 $$ \mathrm{GDP}\ \mathrm{Cost}\left({LR}_{\mathrm{Basel}\_\mathrm{III}}\right)=\left({R}_f+\left(\frac{\widehat{a}\bullet {LR}_{\mathrm{Basel}\_\mathrm{III}}}{C_{\mathrm{con}}}+\widehat{b}\right)\bullet {R}_p\right)\bullet \frac{E_{Y, PK,t}\bullet SEF\bullet PT}{P_{K,t}} $$

After defining a base level of *LR*_Basel _ III _ 0_ as a point of departure for the increases of the leverage ratio, *LR*_Basel _ III_, we can simplify the equation as

12 $$ \mathrm{GDP}\ \mathrm{Cost}\ \mathrm{Line}\ \left({LR}_{\mathrm{Basel}\_\mathrm{III}}\right)={R}_p\ \frac{E_{Y, PK,t}\bullet SEF\bullet PT}{P_{K,t}}\left(\frac{\widehat{a}\bullet {LR}_{\mathrm{Basel}\_\mathrm{III}}}{C_{\mathrm{con}}}-\frac{\widehat{a}\bullet {LR}_{\mathrm{Basel}\_\mathrm{III}\_0}}{C_{\mathrm{con}}}\right)\kern0.75em $$

Equation (12) is a linear, upward-sloping function of the leverage ratio and measures the GDP cost of additional capital requirements in comparison to a given base level of LR. We use this equation to calculate the GDP impact of higher capital requirements.

For the base level for *LR*_Basel _ III _ 0_ here and for the GDP benefit curve developed in the next section, we select 3.3%, which is approximately the mean value of the Basel III converted leverage ratio for Tier1 over the period 2013 to 2015. Inserting the already mentioned values of the parameters (see Appendix 3, Tables 14 and 15 for a detailed presentation of the parameters and their values) into Eq. (12) allows us to calculate the social economic cost resulting from a 1 percentage point increase in the leverage ratio. Table 4 presents the results.

**Table 4:** Impact on real GDP resulting from a 1 percentage point increase in the Basel III leverage ratio assuming 100% pass through

Increase in LR	Linear regression (1)		No M-M effect (2)	
	Impact on WACC (RP = 5%)	Impact on WACC (RP = 10%)	Impact on WACC (RP = 5%)	Impact on WACC (RP = 10%)
Basel III Tier 1 Look-through	0.030%	0.032%	0.055%	0.060%
Basel III CET1 Look-through	0.038%	0.042%	0.071%	0.077%

Note: (1) The calculation is based on the estimated M-M offset resulting from the linear regression 2001Q2 to 2015Q2 (see Table 1). (2) Calculated under the assumption that the required return remains invariant to leverage

Table 4 shows the results assuming a 100% pass through. The social economic costs related to higher capital requirements for the Swiss G-SIBs are very small. A 1 percentage point increase in the TBTF leverage ratio for Basel III Tier1 capital leads to a permanent annual output losses of 0.03% to GDP. In terms of the Basel III CET1 leverage ratio, the impact is slightly stronger with a permanent fall in the level of real GDP by 0.04%. Using an annual discount rate of 5%, the estimates imply a fall in the present value of all future GDP of between 0.6 and 0.8%.^24^ Thus, the recent decision by Switzerland to lift the TBTF Basel III Tier1 LR from 3 to 5% implies a social economic cost of about 0.06% per annum whose present value is equal to 1.2% of current output. Note that the size of the market risk premium does not matter very much for the results in Table 4. It influences the economy as a whole (both the banking and the corporate sector simultaneously) leaving the relative cost between the banking and the corporate sector largely unaffected.^25^

The last two columns of Table 4 report the social economic costs if there were no M-M offset. They are nearly twice as high as the results which include the M-M effect, thereby once more highlighting that the M-M effect matters.

Finally, if we assume a pass through of 50%, then all reported values are simply halved and—as we will see below—the optimal leverage ratio becomes larger.

## Social benefits of additional capital requirements

In this section, we provide an updated estimate of the effects of banking crises on the growth path of Swiss GDP. It is based on the analysis of Junge and Kugler (2013) who use data from 1881 to 2010. In the interim, however, we have better historical data for real GDP from 1892 to 1947 available and do not have to deflate nominal GDP by the consumer price index in order to get a proxy for real GDP for the years before 1948. Moreover, we now have nearly 10 years of data since the last financial and banking crisis and we should therefore get a much more reliable estimate of its effect on GDP.

Switzerland has experienced four fully fledged banking crises since 1881, namely in 1911, 1931, 1991, and 2007.^26^ In addition, we account for the recessions of the two world wars (1916 and 1942) as well as the oil price shock of 1974. In order to estimate the long-run impact of these crises, we use a deterministic time trend model for log GDP taking into account the effects of major shocks by including level shift dummy variables (being equal to 0 before the event and 1 after) for all major adverse shocks. The dummies do not capture the short-run effect of a crisis but only their permanent effects on GDP. Thus, the results are robust and minor differences of plus or minus 1 year in dating the crises do not matter. The transitory cyclical deviations from the trend are captured by the residual of Eq. (13) which we expect to be strongly autocorrelated but stationary.

13 $$ \log (GDP)={\gamma}_0+{\gamma}_1t+{\delta}_1D{1911}_t+{\delta}_2D{1931}_t+{\delta}_3D{1991}_t+{\delta}_4D{2007}_t+{\delta}_5D{1916}_t+{\delta}_6D{1942}_t+{\delta}_7D{1973}_t+{\varepsilon}_t\kern40.75em $$

Before turning to the results of this model, let us briefly mention that the residuals of this deterministic trend model appear to be stationary. Indeed, the residuals are identified as following an AR(1) process with a coefficient of 0.65 and a Kwiatkowski-Philips-Schmidt-Shin (KPSS) test does not reject at any reasonable significance level the null hypothesis of stationarity (KPSS = 0.0758, 10% critical value = 0.119). However, the standard critical values are not valid for residuals of trend break models. In order to get the appropriate critical values, we ran 1000 bootstrap replications taking into account the AR(1) property of the residuals. By this exercise, we obtained 10%, 5%, and 1% critical values of equal to 0.145, 0.169, and 0.212, respectively. Thus, the stationarity hypothesis is clearly in line with the data, as the KPSS statistic calculated is lower than the appropriate 10% critical value of 0.145.

The empirical results for this model and annual Swiss data from 1892 to 2016 are presented in Table 5. First of all, consider the coefficient estimate for the time trend. It is 0.0344, which implies a potential GDP growth of nearly 3.5% instead of the historical average of 2.34%. This drop in measured GDP growth was brought about by permanent shifts of the GDP growth path by the crises reflected in our dummy variables.

**Table 5:** Trend model for Swiss GDP and the effects of big crises, 1892–2016

Regressor	Coefficient estimates, unrestricted	Coefficient estimates, restricted
*T*	0.0339 (0.00285)^***^	0.0344 (0.00238)^***^
*D1911*	− 0.0810 (0.0387)^**^	− 0.196 (0.0431)^***^
*D1931*	− 0.250 (0.0714)^***^	− 0.285 (0.0595)^***^
*D1991*	− 0.298 (0.0811)^***^	− 0.285 (0.0595)^***^
*D2007*	− 0.193 (0.0649)^***^	− 0.196 (0.0431)^***^
*D1916*	− 0.252 (0.0769)^***^	− 0.109 (0.0434)^*^
*D1942*	− 0.0621 (0.0723)	− 0.109 (0.0434)^*^
*D1974*	− 0.107 (0.0806)	− 0.109 (0.0434)^*^
Adjusted *R*^2^	0.990	0.989
Standard error of residual	0.0876	0.0912
Durbin-Watson statistics	0.699	0.561
Number of observations	125	125
*F*-test: *δ*_1_ = *δ*_2_ = *δ*_3_ = *δ*_4_ = *δ*_5_ = *δ*_6_ = *δ*_7_	4.379^***^	
*F*-test: *δ*_1_ = *δ*_2_ = *δ*_3_ = *δ*_4_	7.734^***^	
*F*-test: *δ*_5_ = *δ*_6_ = *δ*_7_	3.241^*^	
*F*-test: *δ*_1_ = *δ*_4_, *δ*_2_ = *δ*_3_, *δ*_5_ = *δ*_6_ = *δ*_7_	2.076^*^	
*δ*_2_ − *δ*_5_		0.176 (0.0665)^***^

Note: ^*^,^**^,^***^Significance at the 5%, 1%, and 0.1% level, respectively

Standard errors corrected for heteroscedasticity and autocorrelation (Newey-West) are given in parentheses percent). Data sources: 1892–2005: Swiss economic and social history online database, Table Q16a, b, http://www.fsw.uzh.ch/hstat/nls_rev/ls_files.php?chapter_var=./q; 2006–2016: https://data.snb.ch/de/topics/uvo#!/cube

We see that, in particular, the occurrence of banking crises has a strong and highly statistically significant permanent negative impact on the level of GDP. For instance, we see that the largest negative impact of approximately 30% is associated with the crisis in the early 1990s (estimate of *δ*_3_ = − 0.298). In general, the results are qualitatively similar to those obtained in Junge and Kugler (2013) using the old data set. However, there are some quantitative differences. We find a statistically different impact for the four banking crises according to the corresponding highly significant *F*-statistic reported in Table 5: The banking crises of 1931 and 1991 clearly had a stronger effect than those of 1911 and 2007. This appears plausible as in 1931 and 1991 the banking crises occurred in connection with a strong depression, or at least a strong recession, whereas in 1911 and 2011 the crises originated in the banking sector.

For the non-banking crises, we also found negative permanent effects, but their impacts are lower and of lesser statistical significance and there is weak evidence that these crises had a different effect, i.e., WW I appears to have had a stronger impact on Swiss GDP than WW II and the oil crisis. Nevertheless, we use the restriction that the three non-banking crises have the same effect along with the assumption of different effects of the two pairs of banking crises. The *F*-statistic for these restrictions is approximately 2, which has a marginal significance level of 9% and cannot be rejected at the usual 5% significance level. The estimation results for this restricted model are provided in the last column of Table 5.

**Fig. 1:**
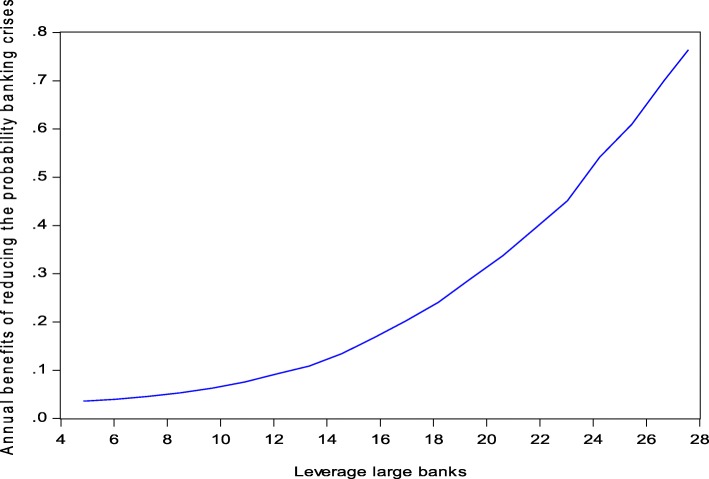
Expected annual GDP benefits and trend leverage of large banks. Source: Junge and Kugler (2013), Figure 8

The estimated long-run impact of a “pure” banking crisis is − 0.196 whereas the recession triggered banking crises have a larger impact estimate of − 0.285. As the effect of the other crises on the growth path of GDP is estimated to be − 0.109, we get a new “indirect” net estimate of a banking crises of 0.176 (the difference between − 0.285 and − 0.109) which is statistically highly significant. Interestingly, the new indirect estimate of the net effect of a banking crisis is almost exactly equal to the one reported in Junge and Kugler (2013). Moreover, the long-run impact on GDP of pure banking crises is estimated as − 0.196. This “direct” estimate is very close to the indirect estimate, and we conclude that we can safely use the benefit function reported in Junge and Kugler (2013), which is displayed in Fig. 1, for the determination of the optimal leverage ratio.

The estimated impact of banking crises on Swiss GDP (Fig. 1) is based on a probit estimate of the dependence of the annual probability of the occurrence of a banking crisis on leverage. The explanatory variables of this model are leverage of the Swiss large banks, interest rate spread (mortgage/savings rate), real GDP growth, and inflation.^27^ For this purpose, we decomposed the first three variables into a transitory or cyclical and a permanent or trend component using the HP filter. Inflation was decomposed into an expected (using an AR(2) model to predict inflation) and an unexpected inflation rate (the residual of the AR(2) model). All regressors were lagged 1 year in order to avoid simultaneity problems. We have to mention that leverage is defined as total assets divided by total book equity. This approach was chosen for data reasons, since it was only for this definition of leverage that we had the long-time series we need for our analysis.

For leverage and the interest rate spread, only the cyclical component was statistically significant. An increase in cyclical leverage (interest rate spread) leads to an increase (decrease) in the probability of a banking crisis. The findings appear reasonable: a strong short-run increase in leverage and a cyclical decline in the interest rate spread are indicators for overexpansion, with fierce competition in the banking sector, and are typical of the euphoria paving the way to a bubble. The change in trend GDP (10% significance) and in expected inflation (5% significance) reduce the probability of a banking crisis. These results were in line with our a priori expectations. An increase in trend growth indicates that loans become less risky and the incomplete adjustment of bank (sight) deposit rates to inflation. There is no direct significant effect of the trend component of leverage on the probability of a banking crisis, but there is an indirect impact resulting from the relationship between the variability of the cyclical component and the trend component of leverage. Indeed, the application of an EGARCH model provided a statistically highly significant effect of trend leverage on the variance of the cyclical leverage component. This function was estimated as the mean of 50,000 Monte Carlo replications simulating the effect of the trend component of leverage on the probability of a banking crisis. This function is finally multiplied by the estimated GDP loss of a banking crisis in order to get the function displayed in Fig. 1. The details of the model estimation are reported in Junge and Kugler (2013).

For further analysis, we follow the approach of Cline (2016) and approximate the function displayed in Fig. 1 by an exponential expression:

14 $$ Expected\  GDP\  benefit\ \left({L}_{\mathrm{Base}\mathrm{l}\_\mathrm{III}}\right)=A\bullet {\left({B}_{\mathrm{con}}\bullet \kern0.5em {L}_{\mathrm{Base}{\mathrm{l}}_{\mathrm{III}}}\right)}^{\rho}\kern9.5em $$

This function provides a very close fit (*R*-squared = 0.998) to the data of Fig. 1 and the exponent *ρ* is estimated to be 2.54 and the constant *A* 1.56E−04. The exponent describes the convex slope of the function and the constant *A* reflects the expected GDP loss when the leverage is zero, i.e., the asset/capital ratio is 1.^28^ Moreover, the function is now expressed in terms of the Basel III leverage. The conversion factor is *B*_con_ = 0.676 (= 0.73/1.08, see Appendix 1) and turns the accounting-based leverage multiple of balance sheet assets/book equity used in the estimation of the probability function into a Basel III compliant expression.

This function is transformed in terms of the leverage ratio LR = 1/*Lev*:

15 $$ Expected\  GDP\  benefit\left({LR}_{\mathrm{Basel}\_\mathrm{III}}\right)=A\bullet {\left({B}_{\mathrm{con}}\bullet \frac{1}{{\mathrm{LR}}_{\mathrm{Basel}\_\mathrm{III}}}\right)}^{\rho }=A\bullet {\left(\frac{B_{con}}{{\mathrm{LR}}_{\mathrm{Basel}\_\mathrm{III}}}\right)}^{\rho}\kern0.5em $$

The change in expected benefits compared to a base leverage ratio *LR*_Basel _ III _ 0_ is therefore given by the following equation:

16 $$ Change\ in\  GDP\  benefit\left({LR}_{Basel\_ III}\right)=A\bullet {B}_{con}^{\rho}\bullet \left(\frac{1}{{\mathrm{LR}}_{\mathrm{Basel}\_\mathrm{III}}^{\rho }}-\frac{1}{{\mathrm{LR}}_{\mathrm{Basel}\_\mathrm{III}\_0}^{\rho }}\right)\kern1em $$

**Fig. 2:**
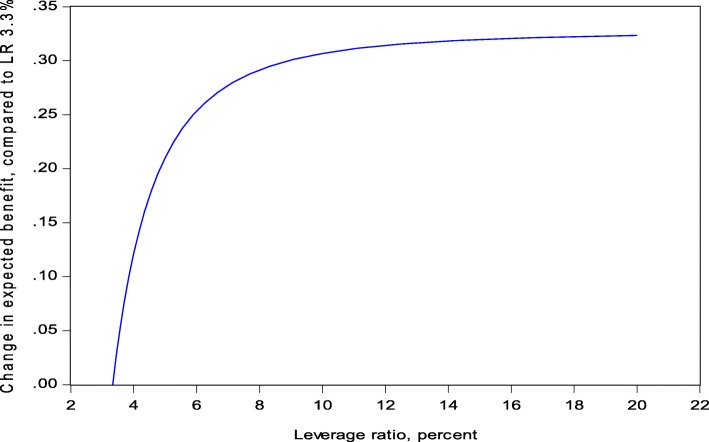
Change in expected annual GDP benefits and leverage ratio of large banks in percent

This function is displayed in Fig. 2 where the starting value of the leverage ratio is set to 3.3%, with the approximate mean value of the Basel III converted leverage ratio expressed in terms of Basel III Tier1 over the period 2013 to 2015.

A 1 percentage point increase of the leverage ratio from 3.3 to 4.3% yields a GDP benefit of 0.16%. This is clearly above the impact on GDP cost of 0.03% (see Table 4) and in line with the conclusion of Junge and Kugler (2013) that the benefits exceed long-run costs by a substantial multiple. However, after a certain level the marginal benefit of additional capital declines and falls short of marginal cost. For example, a 1 percentage point increase of the leverage ratio from 7 to 8% amounts to only 0.01% GDP benefit and hence is below GDP cost. This behavior stems directly from our estimation of the annual crisis probability and reflects the fact that extreme crisis events are rare and require significantly more capital.

The sharply shaped benefit curve is an observation that has also been made in other studies. We have already mentioned Cline (2016). But Miles et al. (2011 and 2013), Brooke et al. (2015) and a recent IMF paper (Dagher et al. 2016) also estimate similar shapes of benefit curves. The common feature is that the marginal benefits of additional capital are material at first, but rapidly decline after a certain level of bank capitalization.

## Comparing social cost and benefits and the determination of the optimal leverage ratio

Using the cost line Eq. (12) and the benefit curve Eq. (16), we calculate the social marginal cost (MC) and benefit (MB) and determine the optimal leverage ratio for the Swiss G-SIBs. The optimal leverage ratio will occur where the two marginal effects are equal (MC = MB).

The derivative of the social cost line Eq. (17) with respect to the required Basel III leverage ratio is:

17 $$ MC={R}_p\bullet GDPM\bullet \frac{\widehat{a}}{C_{\mathrm{con}}}\kern2.5em $$

All terms in Eq. (17) are constants and hence the derivative with respect to the leverage ratio is a constant.

The derivative of the benefit Eq. (16) is:

18 $$ MB=\gamma \bullet A\bullet {B}_{\mathrm{con}}^{\rho}\bullet {LR}^{-\uprho -1}\kern0.5em $$

Equation (18) states that increases of the leverage ratio reduce the marginal benefit. The shape of the function is concave and reflects the diminishing benefit to increases in the leverage ratio.

Solving for the optimal LR* yields:

19 $$ {\mathrm{LR}}^{\ast }={\left(\frac{\rho \bullet A\bullet {B}_{\mathrm{con}}^{\rho }}{R_p\bullet GDPM\bullet \frac{\widehat{a}}{C_{\mathrm{con}}}}\right)}^{\frac{1}{1+\rho }}\kern0.5em $$

**Fig. 3:**
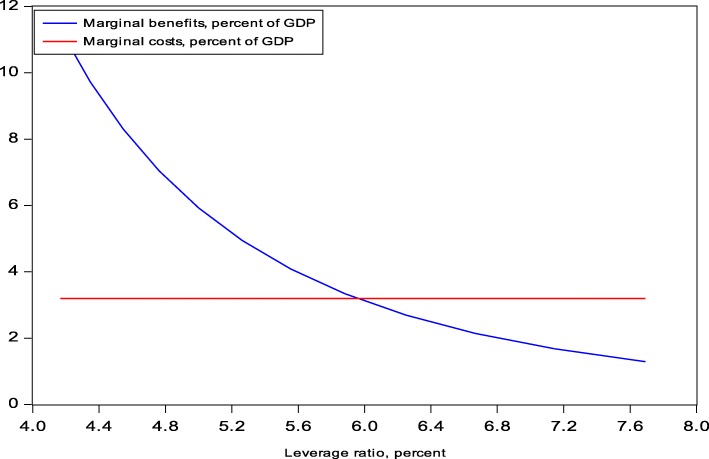
Optimal Leverage Ratio LR*, Basel III Tier1, PR=5%

Table 6 reports the base case for the optimal LR* for Swiss G-SIBs in terms of the Basel III Tier1 and CET1 leverage ratios, and Fig. 3 provides the graphical presentation in terms of Basel III Tier1. The base case varies with respect to two parameters: the capital definition (Basel III Tier1 or CET1) and the Risk Premium (5% or 10%).^29^

**Table 6:** Base case: optimal TBTF leverage ratios for Swiss G-SIBs

Optimum and minimum required LR	Basel III Tier 1 (%)	Basel III CET1 (%)
LR* (RP = 5%)	6.07	4.43
LR* (RP = 10%)	5.93	4.33
Minimum required LR	5.00	3.50

The base case suggests that the optimal leverage ratio for Basel Tier 1 capital requirements is about 6% and for CET1 capital requirements about 4.4%. Thus, the Swiss regulatory TBTF minimum leverage ratios fall short of the optimal level by about 1 percentage point. This result can be translated into risk-weighted capital ratios. Since the Swiss TBTF framework establishes a fixed linear relationship between the leverage ratio and the capital ratio for Swiss G-SIBs,^30^ capital ratios are easily calculated and compared to other studies of optimal capital ratios (see Table 7).

**Table 7:** Optimal capital ratios and minimum equity requirements

Mainly large systemically important banks		Source	Basel III Tier1	Basel III CET1
Optimal capital ratios	Switzerland	Junge and Kugler (2013)	17%	12.5%
	UK	Miles et al. (2011 and 2013)	20%	
		Brooke et al. (2015)	10–14%	
	Sweden	Sveriges Riksbank (2011)		14–17%
	Norway	Norges Bank (2012)		16–23%
	Industrial countries	BCBS (2010a)		12.5%
		Dagher et al. (2016, IMF)	15–23%	
		Cline (2016)		11.7–14.1%
Minimum equity requirements		Switzerland	14.3%	10.0%
		BCBS	9.5–11.0%	8.0–9.5%

Going over Table 7 leads to three conclusions: First, the optimal capital ratios for Swiss G-SIBs are about 2.5 percentage points higher than the required Swiss TBTF capital ratios of 14.3% (Basel III Tier1) and 10% (CET1). Second, a similar picture emerges for the large banks of other countries. The optimal capital ratios are always above the minimum equity requirements of the BCBS. Third, results vary across studies. With the exception of Brooke et al. (2015), all studies estimate optimal capital ratios above 15%. This difference mainly results from a different understanding of loss-absorbing bank capital. As in all the other studies, Brooke et al. (2015) estimate the optimal capital ratios on the basis of going concern equity capital because bank equity is the only reliable loss absorber in financial crises. Other forms of capital, in particular hybrid capital, failed in the financial crisis of 2007/2008. Nevertheless, Brooke et al. (2015) make a general downward adjustment of their optimal capital ratios to reflect the regulatory requirement that banks have to meet gone concern capital in addition to the going concern bank equity.^31^ Gone concern capital is equity-like capital such as subordinated debt subject to bail-in. Its intention is to provide capital for failed banks in order to recapitalize, restructure, or wind them down without using taxpayer funds. Moreover, from a conceptional point of view, it is not appropriate to mix going and gone concern capital considerations as it would imply not only a different cost curve but also a benefit curve that includes two objectives: (i) the reduction of crisis probability due to higher equity capital and (ii) the benefits of an orderly resolution due to gone concern capital.

Interestingly, dynamic general equilibrium models exhibit similar results as presented in Table 7. Clerc et al. (2015) set up a dynamic stochastic general equilibrium model which allows an explicit welfare analysis of macroprudential policies. Similar to the approaches reported above, increases in capital requirements imply both a reduction in the supply of loans due to higher interest rates and a lower average default rate of banks. The authors point out that the steady-state solution of their model is consistent with the results of Miles et al. (2011 and 2013) and BIS (2010).^32^ The dynamic equilibrium model of Martinez-Miera and Suarez (2014), which also includes an explicit welfare analysis, points in the same direction. They find a socially optimal CET1 capital ratio of 14%.

It is reassuring to see that alternative approaches yield similar results. Nevertheless, it is useful to assess the uncertainty attached to the estimations and the choice of parameters. We thereby follow a methodology of Cline (2016) and provide alternative parameter values for key variables and calculate optimal LRs* for all possible combinations. The parameter values considered are reported in Table 15 in Appendix 3 together with a detailed account of the selection of the different parameter values. In general, we use plus minus two standard errors around an estimated parameter value if this approach is possible. The table also includes alternative M-M offsets and pass through scenarios.

**Fig. 4:**
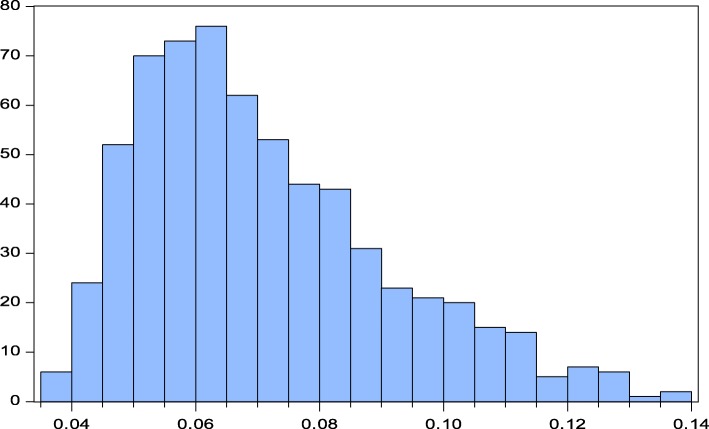
Histogram of Basel III Tier1 Optimal Leverage Ratio LR*

**Fig. 6:**
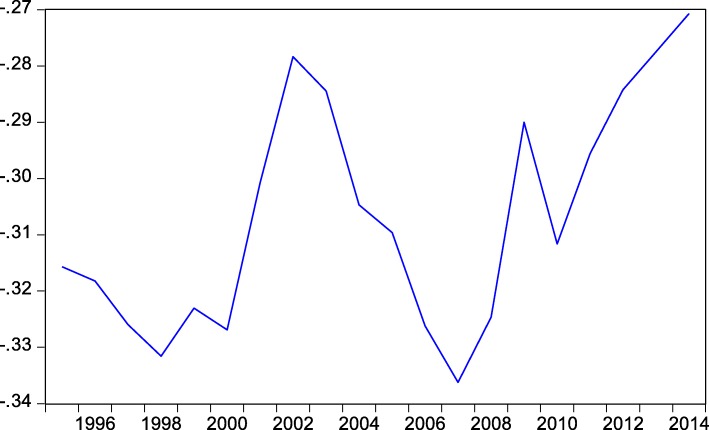
Translog estimate of the elasticity of GDP to the price of capital, Switzerland 1995-2014

Combining the values reported in Table 15 results in 648 combinations of parameter values.^33^ Figure 4 presents a histogram of these calculations. The lowest optimal LR* is 3.72%, which is obtained by assuming a zero M-M offset (*â* = 1), the higher risk premium (10%), a larger share of external financing of firms affected by a capital cost increase (18.5%), a high elasticity of GDP with respect to capital costs (0.34), a lower GDP loss severity (10%), and a downward adjusted exponent of the benefit curve (minus 2 standard errors). The median of the optimal LR* is 6.67% which is slightly above the benchmark case. The maximum LR* is equal to 13.69% which emerges by assuming an M-M offset of 67%, a low risk premium (5%), the share of non-financial corporates’ financing provided by G-SIBs at a reduced level (5.4% percent), a low elasticity of GDP with respect to capital costs (0.27), a high GDP loss severity (28.5%), and an adjusted exponent of the benefit curve (plus 2 standard errors). It is worth mentioning that the asymmetry of the frequency distribution is strongly driven by the M-M offset and the pass through.

Finally, the CET1 histogram exhibits a similar pattern as shown for Basel III Tier1 in Fig. 4. Its median is equal to 4.86%, which is above the minimum TBTF standard of 3.5% and somewhat above the optimal base case LR*of 4.43% of the CET1 base case. When we vary the parameter values, we arrive at a minimum optimal CET1 leverage ratio of 2.91 and a maximum of 9.99%, respectively.

## Conclusions

This paper extends the analysis of Junge and Kugler (2013) on the effects of increased equity capital requirements on Swiss GDP to the determination of an optimal leverage ratio. In addition, we improved our model by using the flexible translog production function (instead of CES), updated our estimates, and used newly available historical GDP data to estimate the effect of a banking crisis on real GDP. The study finds that the optimal leverage ratios for Swiss G-SIBs are of approximately 6% in terms of Basel III Tier1 and 4.5% in terms of CET1. The corresponding optimal risk-weighted capital ratios are 17% and 13%, respectively. On this basis, the revised minimum TBTF requirements for the Swiss G-SIBs fall short of optimal leverage and capital ratios by about 20%.

The paper also addresses the large range of uncertainty surrounding the estimates. Although variations in the key parameters can result in big changes in the estimated optimal capital requirements ranging from 3.7 to 13.7%, the median of the distribution is 6.7%, which is slightly above our benchmark estimate of 6.1%. The minimum value is mainly due to the assumption of no M-M offset, a one-to-one pass through of interest rate adjustment of G-SIBs and relatively low GDP loss of a banking crisis. By contrast, the maximum of 13.7% for the leverage ratio is based on a 67% M-M offset, a 50% interest rate pass through, and a high GDP loss of a banking crisis.

Our estimates of optimal equity requirements are smaller than the Admati and Hellwig (2013) proposition of 20 to 30%. Their argument is based on the full M-M offset that higher equity capital requirements would not increase the banks’ overall funding costs and hence do not impact GDP. In our model, this assumption, strictly speaking, leads to an undetermined optimal leverage ratio. Optimal leverage ratios of the order of 20 to 30% imply a nearly complete M-M offset and/or very low interest rate pass through resulting in a downward shift of the marginal cost curve. Besides these two determinants of the optimal leverage ratio, the limiting factor for additional increases in capital requirements stems mainly from the GDP benefit curve. Its shape implies that the marginal benefits of additional capital decline sharply at leverage ratios clearly below 20–30%.

Finally, given the uncertainty around our estimates, we are the first to caution against a too-literal interpretation of the “optimal” equity capital requirements. Rather, our investigation of the trade-off between social cost and social benefit of higher equity capital requirements should be taken as an important complementary alternative to other approaches to bank capital determination. At any rate, our investigation addresses the central question of the optimal level of bank equity capital. The issue, however, is far too complex to be treated by one approach alone. Instead, different approaches—including international benchmarking exercises and competitiveness considerations as applied by the Swiss Group of Experts—should be used to determine the appropriate level of bank equity.

## Figures and Tables

**Fig. 5:**
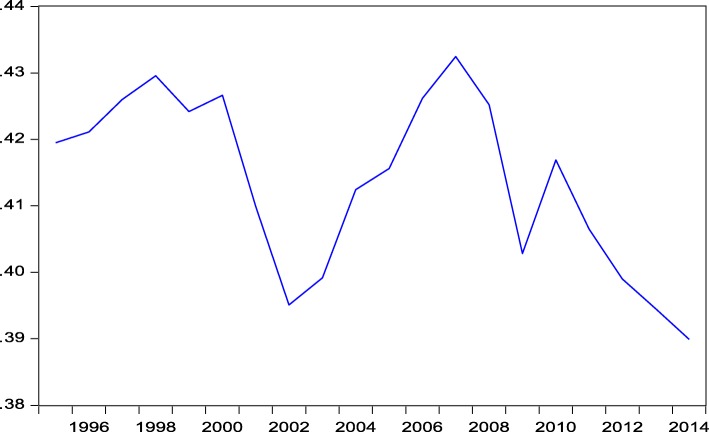
Cost share of capital, Switzerland 1995-2014. Data source: Capital and Labour income, employment and hours worked, https://data.snb.ch/de/topics/uvo#!/cu. Capital stock, https://www.bfs.admin.ch/bfs/de/home/statistiken/volkswirtschaft/kapitalstock.html

## Appendix 1

### Regulatory capital definitions and conversion methods

This Appendix presents the various definitions of leverage ratios used to calculate the economic costs and benefits of higher equity capital requirements and explains how they can be converted into a common leverage ratio in line with the definitions of the Basel III Accord. Based on this conversion, we are able to express our results in terms of the Basel III definition of the leverage ratio.

The estimation of the M-M offset and *WACC* before any conversion applies the Basel II BIS Tier1 capital definition as numerator and the banks’ Balance Sheet Asset as denominator of the leverage ratio. The estimation of the annual probability of banking crises occurring, and the economic benefit before conversion, are estimated using Book Equity defined as capital and Balance Sheet Assets as the denominator in the leverage ratio.

$$ \mathrm{Simple}\ \mathrm{leverage}\ \mathrm{ratio}=\frac{\mathrm{Basel}\ \mathrm{II}\ \mathrm{BIS}\ \mathrm{Tier}1\kern0.5em \mathrm{Capital}\ \left(\mathrm{Cost}\right)\ \mathrm{resp}.\mathrm{Book}\ \mathrm{Equity}\ \left(\mathrm{Benefit}\right)\kern1em }{\mathrm{Balance}\ \mathrm{Sheet}\ \mathrm{Assets}\ } $$

In order to compare the results of various definitions of the leverage ratio, the latter must be made compatible with a common Basel III basis.

The new Basel III definition requires that the numerator consists of loss-absorbing equity capital, i.e., dominantly CET1 and a proportion of AT1. This is a markedly stricter definition than the Basel II BIS Tier1 capital definition. In particular, the Basel III definition excludes any hybrid capital items, which were found in the financial crisis to be poor in absorbing losses. Also the definition of the denominator of the Basel III leverage ratio (LRD) goes beyond the definition of balance sheet assets. It additionally includes off-balance sheet items and treats the calculation of securities financing transactions and derivatives in its own way.^34^

In order to convert the different capital and asset definitions to the Basel III standards, we used the leverage ratios reported by CS and UBS under both a Basel II and Basel III approach for a common reporting period.

Tables 8, 9, 10, 11, and 12 present the results. All conversion factors refer in each case to the look-through or fully-applied equity capital definition of Basel III. They capture the equity capital position of the banks assuming the full application of Basel III, excluding the phase-in adjustment of the transition period from 2014 up to 2018. The conversion factors related to CET1 and Basel II BIS Tier1 and between CET1 and Book Equity are shown in Tables 8 and 9, respectively, and were calculated on the basis of a common (pre-phasing-in) reporting period from Q4 2011 to Q4 2013.

$$ \mathrm{Basel}\ \mathrm{I}\mathrm{I}\mathrm{I}\ \mathrm{CET}{1}_{\mathrm{Look}-\mathrm{through}}=0.60\times \mathrm{Basel}\ \mathrm{I}\mathrm{I}\ \mathrm{BIS}\ \mathrm{Tier}1 $$

$$ \mathrm{Basel}\ \mathrm{III}\ \mathrm{CET}{1}_{\mathrm{Look}-\mathrm{through}}=0.52\times \mathrm{Book}\ \mathrm{Equity} $$

**Table 8:** Conversion factor: Basel III CET1 Look-through to BIS Basel II Tier1

Basel III CET1_Look-through = 0.60 * BIS Basel II Tier1					
Period	CS		UBS		Combined
	Basel II BIS Tier1Mio CHF	Basel III CET1Look-through Mio CHF	Basel II BIS Tier1Mio CHF	Basel III CET1Look-through Mio CHF	Conversion factor Basel III CET1/Basel II Tier1
2011Q4–2013Q4	43′730	23′895	40′406	26′902	0.60

Sources: BIS Tier1 quarterly observations gathered via Bloomberg and quarterly financial reports of CS and UBS. CET1 Look-through respectively CET1 Fully-applied: Quarterly Financial Reports of CS and UBS. The pro-forma CET1 Look-through figures of CS for Q2 2012 and 2013 and for Q4 2012 were collected from CS Investor Day Presentations, in particular: Barclays Global Financial Services Conference, September 12, 2012, and September 11, 2013

**Table 9:** Conversion factor: Basel III CET1 Look-through to Book Equity

Basel III CET1_Look-through = 0.52 * Book Equity					
Period	CS		UBS		Combined
	Book equity Mio CHF	Basel III CET1 Look-through Mio CHF	Book equity Mio CHF	Basel III CET1 Look-through Mio CHF	Conversion factor Basel III CET1/Book Equity
2011Q4–2013Q4	45′241	23′895	51′802	26′902	0.52

Sources: Book Equity: Quarterly observations gathered via Bloomberg. CET1 Look-through respectively CET1 Fully-applied: Quarterly Financial Reports of CS and UBS. The pro-forma CET1 Look-through figures of CS for Q2 2012 and 2013 and for Q4 2012 were collected from CS Investor Day Presentations, in particular: Barclays Global Financial Services Conference, September 12, 2012 and September 11, 2013

**Table 10:** Conversion factor: Basel III Tier1 Look-through to Basel II Tier1

Basel III Tier1_Look-through = 0.77 * BIS Basel II Tier1					
Period	CS		UBS		Combined
	Basel II BIS Tier1 Mio CHF	Basel III Tier1 Look-through Mio CHF	Basel II BIS Tier1 Mio CHF	Basel III Tier1 Look-through Mio CHF	Conversion factor Basel III Tier1/Basel II Tier1
2013Q4–2015Q3	46′864	38′207	42′115	31′622	0.77

Sources: BIS Basel II Tier1: Quarterly observations gathered via Bloomberg. Basel III Tier1 Look-through respectively CET1 Fully-applied: Quarterly Financial Reports of CS and UBS

**Table 11:** Conversion factor: Basel III Tier1 Look-through to Book Equity

Basel III Tier1 Look-through =0.73 * Book Equity					
Period	CS		UBS		Combined
	Book equity Mio CHF	Basel III Tier1 Look-through Mio CHF	Book equity Mio CHF	Basel III Tier1 Look-through Mio CHF	Conversion factor Basel III Tier1/Book Equity
2013Q4–2015Q3	44′584	38′207	53′032	31′622	0.73

Sources: Book Equity: Quarterly observations gathered via Bloomberg. Basel III Tier1 Look-through respectively CET1 Fully-applied: Quarterly Financial Reports of CS and UBS

**Table 12:** Conversion factor: ratio LRD to Balance Sheet Assets

Basel III LRD Look-through = 1.08 * Balance Sheet Assets					
Period	CS		UBS		
	Balance Sheet Assets US GAAP in Mio CHF	Basel III LRD Look-through in Mio CHF	Balance Sheet Assets IFRS in Mio CHF	Basel III LRD Look-through in Mio CHF	Conversion factor LRD/Balance Sheet Assets
2014Q4–2015Q3	890′899	1′089’770	1′010’311	966′414	1.08

Sources: UBS: Quarterly Reports 2014 and 2015, Section: Capital Management. CS: Quarterly Reports 2014 and 2015, Section: Treasury, Risk, Balance Sheet and Off-Balance Sheet

In the same way, we calculated the conversion factors between Basel III Tier1 Look-through and *BIS Basel II Tier1* and Book Equity (see Tables 10 and 11). The conversion factors were determined as follows:

$$ \mathrm{Basel}\ \mathrm{I}\mathrm{I}\mathrm{I}\ \mathrm{Tier}{1}_{\mathrm{Look}-\mathrm{through}}=0.77\times \mathrm{BIS}\ \mathrm{Basel}\ \mathrm{I}\mathrm{I}\ \mathrm{Tier}1 $$

$$ \mathrm{Basel}\ \mathrm{III}\ \mathrm{Tier}{1}_{\mathrm{Look}-\mathrm{through}}=0.73\times \mathrm{Book}\ \mathrm{Equity} $$

Again, the calculations are based on the quarterly financial reports of CS and UBS. However, for the relationship Basel III Tier1 Look-through versus Basel II Tier1 and Book Equity, we used the data between 2013 Q4 and 2015 Q3 because the banks did not disclose Basel III Tier1 Look-through calculations prior to Q4 2013.

Finally, we calculated the conversion factor between Balance Sheet Assets and the *Basel III* LRD over the period from Q4 2014 to Q3 2015 (Table 12). This is the earliest period available where the two big banks recorded simultaneously LRD and Balance Sheet Assets. The individual conversion factors of the two banks are rather different and reflect to a great deal the differences in the accounting standards of the two banks. CS balance sheet calculations follow US-GAAP while the UBS calculations are based on IFRS standards. Given the differences in the treatment of derivatives and SFTs between US-GAAP and IFRS accounting rules, it is no surprise that the conversion factor of CS is considerably larger than the UBS conversion factor.^35^

The combined CS and UBS conversion factor is:

$$ Basel\  III\  LRD=1.08\times \mathrm{Balance}\ \mathrm{Sheet}\ \mathrm{Assets} $$

It may be objected that the sample is too small to be able to calculate reliable conversion factors. However, there are reasons to believe that the calculated conversion factors are robust. First, a great proportion of on-balance sheet items are treated in the same way across US-GAAP, IFRS, and LRD and hence limit the scope for unfounded measurement deviations. Second, thanks to pro-forma LRD calculations of UBS back to Q4 2012 we can calculate the conversion factor for this period. It typically hovered in a small corridor slightly below 1 with an average conversion factor of 0.98. This suggests that the sampled conversion ratios between LRD and the accounting measurements IFRS and US-GAAP, respectively, are reliable.

## Appendix 2

### Estimation of the translog production function, Switzerland 1995–2014

The translog analysis is usually done in the dual framework of cost-share equations. The term dual means in this context that all the information needed to obtain the relevant parameters of the production function is contained in the corresponding cost function and vice versa. In a model with two production factors *K* and *L*, their corresponding shares in total production costs (*S*_*K*_ and *S*_*K*_) are represented as linear function of factor prices (*P*_*K*_ and *P*_*L*_):

$$ {S}_{K,t}={\delta}_K+{\gamma}_{KK}\log \left({P}_{K,t}\right)+{\gamma}_{KL}\log \left({P}_{L,t}\right)+{\gamma}_{Kt}t(20) $$

$$ {S}_{L,t}={\delta}_L+{\gamma}_{LK}\log \left({P}_{K,t}\right)+{\gamma}_{LL}\log \left({P}_{L,t}\right)+{\gamma}_{Lt}t(21) $$

For theoretical reasons, the *γ* – matrix is symmetric (*γ*_*KL*_ = *γ*_*LK*_) as substitution of capital by labor is symmetric. As the left-hand variables are shares the slope coefficients add up to zero (*γ*_*KK*_ + *γ*_*KL*_ = 0; *γ*_*KL*_ + *γ*_*LL*_ = 0; *γ*_*Kt*_ + *γ*_*Lt*_ = 0), whereas the intercepts add up to 1 (*δ*_*K*_ + *δ*_*L*_ = 1). Given these restrictions, we only have to estimate one equation. Using the restriction *γ*_*KK*_ =  − *γ*_*KL*_ we can write the first equation of the system above as:

$$ {S}_{K,t}={\delta}_K+{\gamma}_{KK}\left(\log \left({P}_{K,t}\right)-\log \left({P}_{L,t}\right)\right)+{\gamma}_{Kt}t(22) $$

Note that this model collapses to the Cobb Douglas case if both *γ* coefficients are zero and we arrive at a constant cost share of capital which is independent of factor price and equal to the intercept term *δ*_*K*_. Correspondingly, the labor cost share is constant and equal to *δ*_*L*_ = 1 − *δ*_*K*_. If the elasticity of substitution is below 1, then we have a positive *γ*_*KK*_ coefficient, and if technical progress is biased in favor of capital, *γ*_*Kt*_ is positive.

The elasticity of substitution is calculated as

$$ {\sigma}_{KL,t}=\frac{\gamma_{KL}+{S}_{K,t}{S}_{L,t}}{S_{K,t}{S}_{L,t}}=\frac{-{\gamma}_{KK}+{S}_{K,t}{S}_{L,t}}{S_{K,t}{S}_{L,t}}(23) $$

which is, of course, 1 for the Cobb Douglas function. As we can see from the equation above, the opposite case of a zero elasticity of substitution implies a maximum positive parameter value of *γ*_*KK*  =  _*S*_*K*_*S*_*L*_.

Figures 5 and 6 display the data used in our estimation. Figure 5 shows the development of the cost share of capital defined as net operating surplus + depreciation (or capital consumption) divided by the sum of capital costs and compensation of employees (labor costs). The factor prices were calculated by dividing capital income by the capital stock and total labor income by the number of hours worked.

The estimation results for the capital share Eq. (22) are given in Table 13. In order to avoid simultaneity problems, we estimated the model with a lag of one for factor prices.

**Table 13:** Estimation results for the translog production function, Switzerland 1995–2014 (standard errors in parentheses)

Parameter	Estimate	Restricted estimate
*γ*_*KK*_	0.1028 (0.04217)	0.1371 (0.03618)
*γ*_*Kt*_	− 0.000554 (0.000513)	–
Adj. *R*^2^	0.3557	0.3637
S.E	0.01084	0.01077
Durbin-Watson	1.2435	1.3461
Number of observations	20	20

Table 13 shows a positive *γ*_*KK*_-estimate which is statistically significantly different from zero and implies a substitution elasticity which is clearly lower than one in absolute value. However, no evidence in favor of a non-neutral technical progress is found, while the deterministic trend coefficient is small and statistically not different from zero. Therefore, we estimated the model without a time trend which gives a slightly higher *γ*_*KK*_-estimate in absolute value. Inserting the time-varying factor shares displayed in Fig. 5 results in an elasticity of substitution estimate varying between 0.42 and 0.44 during the period 1995–2014. Given this time series of the capital cost share (*S*_*K*, *tt*_) and the elasticity of substitution (*σ*_*KL*, *t*_), we are able to calculate a time-varying estimate for the elasticity of production with respect to the price of capital as given in Eq. (9). It varies between − 0.34 and − 0.27 with a mean and median approximately equal to − 0.31. As shown in Fig. 6, the elasticity of output with respect to price of capital reached its absolute maximum before the financial crisis and decreases in absolute value since 2008 implying a weaker reaction of GDP to capital costs changes in recent years.

## Appendix 3

### Parameter values for calculation of the optimal leverage ratios

Tables 14 and 15 list the parameters and the values used in the calculation of the optimal leverage ratio for Basel III Tier1 and CET1 capital.

**Table 14:** Base case parameter values and alternatives

Parameter	Description	Base case	Low opt. LR	High opt. LR
M-M offset	Variation of M-M offset: base case, no M-M offset and 67% offset corresponding to minus two standard errors around an estimated parameter value	46%	0%	67%
*R*_*p*_	Equity risk premium	5%/10%	10%	5%
*SEF*1PT (full pass through)*	Share of non-financial corporates’ financing provided by G-SIBs	10.8%	18.5%	10.8%
*SEF*0.5PT (half pass through)*	Share of non-financial corporates’ financing provided by G-SIBs	5.4%	9.4%	5.4%
*E*_*Y*, *PK*_	Elasticity of production with respect to the price of capital	0.31	0.34	0.27
*A*	Constant of GDP benefit curve and varies with GDP losses of 17.7% (base case), 10% (low LR*) and 28.5% (high LR*)	1.56E−04	8.81E−05	2.51E−04
*ρ*	Exponent of GDP benefit curve	2.541	2.463	2.619

Explanation of the alternative parameter values:

M-M offset variation: base case 46% offset corresponding to *â* = 0.8269, of *â* = 1 (no M-M), *â* − 2 standard errors 0.5059 (M-M offset is 67%)

Risk premium: lower (5%) bound and upper (10%) bound to take care of the variations of equity risk premiums

*SEF*1PT* (full pass through): The lower bound of SEF (10.8%) is the share of G-SIBs in external financing of the Swiss corporate sector. The upper bound includes in addition the Swiss D-SIBs Raiffeisenbank and ZKB (The PostFinance, also a D-SIB, is not allowed to provide loans). The market share of Raiffeisenbank in Swiss domestic loans is 13.5% (See SNB Statistik: Bankenstatistisches Monatsheft, Kreditvolumenstatistik.) for period 2012 to 2015 and that of ZKB is 8% (see ZKB: https://www.zkb.ch/media/dok/corporate/medien/praesentation-rudolf-sigg.pdf). These shares must be multiplied with the share of external financing of the corporate sector (35%) and added to 10.8% in order determine the upper bound of SEF of 18.5%.

*SEF*0.5PT* (half pass through): A half pass through reduces the impact of the average lending rate by 50%.

Production elasticity with respect to the price of capital: The estimations of the translog framework showed that the elasticity varies between 0.27 and 0.34 with a mean and median of 0.31.

*A* is the constant of the benefit curve and defined as crises probability * GDP loss. The base case GDP loss (17.7% of GDP) and the upper GDP loss (28.5% of GDP) are derived from our historical regression analysis (equation 18). The lower GDP loss of 10% is the ratio of the bank losses of CS and UBS (nearly CHF 60 bn.) to GDP in the financial crisis from 2007/2008.

Exponent *ρ*: estimate ± 2 standard errors, 2.463 and 2.619

**Table 15:** Other parameters used to calculate GDP cost and benefit

Parameter	Description	Value
*R*_*f*_	Risk-free money market rate	1%
$\widehat{b}$	Slope coefficient of M-M regression (2001Q2-2015Q2)	0.01754
${\beta}_t^{\mathrm{Corp}}$	Swiss corporate companies	1.1
*Tier1, C*_con_	Leverage ratio: Basel III Tier1 Look-through to Basel II Tier1	0.713
*Tier1, B*_con_	Leverage ratio: Basel III Tier1 Look-through to Book Equity	0.676
CET1*, C*_con_	Leverage ratio: Basel III CET1 Look-through to Basel II Tier1	0.556
*CET1, B*_con_	Leverage ratio: Basel III CET1 Look-through to Book Equity	0.481

## Footnotes

1 Junge and Kugler (2013)

2 See for example the comments of Sergio Ermotti 2017, UBS CEO, in the Neue Zürcher Zeitung (January 7, 2017)

3 See for example the speech of Stefan Ingves (2017), Chairman of the Basel Committee and Governor of Sveriges Riksbank

4 FINMA (Swiss Financial Market Supervisory Authority FINMA) 2015a.

5 Group of Experts Report (2014)

6 Schweizerische Eidgenossenschaft (2016)

7 Switzerland’s domestic systemically important banks (D-SIBs) do not come within the scope of our examination because these banks are not publicly traded on the Swiss stock exchange and therefore cannot be included in the methodological approach pursued in this paper. The three Swiss D-SIBs are Raiffeisen Gruppe, Zürcher Kantonalbank, and PostFinance. The Swiss TBTF legislation requires that D-SIBs have to meet a lower minimum going concern leverage ratio of 4.5%. Note that in contrast to Credit Suisse and UBS, the three Swiss D-SIBs have comfortable levels of capital.

8 Another strand of the literature (e.g., Clerc et al. (2015), Martinez-Miera and Suarez (2014)) uses dynamic general equilibrium models to estimate the trade-off between social benefits and social costs of changes in capital requirements.

9 As a numerical example, assume a bank with a CHF 100 balance sheet financed by CHF 97 in debt and CHF 3 in equity. The return on debt is assumed to be 5% and the return on equity 25%. The overall funding costs are 5.6% [= (5% × 0.97) + (25% × 0.03)]. If the bank decides to raise equity to CHF 5 and reduce debt to CHF 95, the bank is less risky than before. Under 100% M-M validity, the required rate on equity will drop from 25 to 21.75% and the required return on debt from 5 to 4.75%. The overall funding cost however remains unchanged at 5.6% [= (4.75% × 0.95) + (21.75% × 0.05)], i. e., there is a 100% M-M offset.

10 See in particular Miles et al. (2011 and 2013) for a presentation of the theoretical basis of Eq. (1)

11 These authors point out that Equation (1) is a variant of the Hamada framework. See Hamada (1969).

12 Bank CDS spreads seem to indicate that the debt of G-SIBs is not risk free. This is a correct observation. However, it should be taken into account that market-based spreads are risk-neutral and overstate real-world default probabilities. But more importantly, Eq. (1) is based on the idea that equity is more risky the higher is leverage and vice versa.

13 The definition of CET1 was introduced with the announcement of the Basel III framework at the end of 2010. A rough estimate of the CET1 capital ratio of the two Swiss G-SIBs can be derived from the Comprehensive Quantitative Impact Study (QIS) of the BCBS (2010b), (December 16, 2010) and Junge and Kugler (2013), footnote 21.

14 For the linear regression, the M-M offset is:

$$ \frac{\partial {\beta}_{\mathrm{equity}}}{\partial Lev}\bullet \frac{Lev}{\beta_{\mathrm{equity}}}=b\frac{Lev}{\beta_{\mathrm{equity}}}=0.01754\frac{40.38}{1.54}=0.460 $$

15 Miles et al. (2011 and 2013) estimate a M-M offset at the order of 70% for UK banks, Cline (2015) finds that the M-M offset amounts to 45% for US banks, and an ECB (2011) suggest a range of M-M offsets between 41 and 72% for 54 large international banks.

16 Leading empirical studies to estimate the M-M offset are Kashyap et al. (2010), Miles et al. (2011 and 2013), Clark et al. (2015). As an alternative, one could test directly the relationship between the required return on bank equity and bank leverage. However, good time series on expected earnings are essential for this, which we could not obtain.

17 One may argue that the importance of mortgage lending in Switzerland justifies a separate treatment of higher capital requirements on residential housing. Such an approach would be indispensable in any disaggregated macroeconomic model in which different kinds of capital (plant and equipment as well as non-residential and residential buildings) are distinguished. However, in the aggregated macroeconomic model used in this paper there is only capital, which is provided by the business sector. Therefore, we implicitly assume that all construction investments are conducted by the business sector. Although this is an overstatement, it is not utterly wrong when we recall that rented and not owned housing is the dominant form in Switzerland.

18 For an introduction to the estimation of translog production functions, see Berndt (1991, Chapter 9).

19 See in particular Smith (2008), Barnes et al. (2008) and Jones (2003)

20 In principle, the Swiss TBTF legislation applies to both the Swiss G-SIBs and the D-SIBs. However, currently, the D-SIBs are out of scope because details of their regulation are still open. Moreover, the Swiss D-SIBs are well capitalized.

21 The share of all Swiss banks in external financing of Swiss companies is 35% and has been stable for years (see Trend (2013). The market share of the two Swiss G-SIBs in domestic lending is 31% (see: SNB, Bankenstatistisches Monatsheft, Kreditvolumenstatistik). Thus, the relevant G-SIBs’ share in external financing of the Swiss corporate sector is 10.8% (= 0.35 × 0.31).

22 See Boot (2000) for a review of the literature on relationship banking.

23 See Staatssekretariat für Wirtschaft (State Secretariat for Economic Affairs) SECO (2017).

24 We use the discount rate of 5% only in order to facilitate the comparison of our results with the results from other studies. In particular, the BCBS tends to present estimates of social economic cost using a discount rate of 5%. The appropriate social discount factor for Switzerland should be much lower.

25 In Eq. (12), the market risk premium enters the numerator through the change in the banks’ funding costs (Eq. (6)) and the denominator through P_*K*_ with little overall impact on real GDP growth.

26 Ritzmann (1973) is a comprehensive reference for the history of Swiss banks. SNB (2007) provides some information on the history of banking crises in Switzerland including the crisis of 1991.

27 The sample period runs from 1906 to 2010.

28 Note that the constant A = 1.56E−04 refers to expected loss (crises probability * 17.7%).

29 A complete list of the parameters and variants is shown in Appendix 3, Table 14.

30 The link between the leverage and the capital ratios for Swiss G-SIBs is the RWA density, which is the average risk weight per unit of exposure for any given bank ($\frac{\mathrm{RWA}}{LRD}\Big)$, where *LRD* is the leverage ratio denominator (see Appendix 1). In order to ensure a coherent interaction between the leverage and the capital ratios, the Swiss TBTF framework requires an RWA density of 35% for G-SIBs. Hence, the capital ratio (CR) is easily determined from the leverage ratio and the RWA density: $CR=\frac{\mathrm{LR}}{\mathrm{RWA}\ \mathrm{dDensity}}$.

31 See Brooke et al. (2015), chart 9 and the critique of Vickers (2016) pointing out that the approach of Brooke et al. (2015) is misguided.

32 See Clerc et al. (2015), page 38

33 We have four parameters that can take three values and three parameters that can two values resulting in a total of 3^4^ times 2^3^ combinations.
